# Scattering Assisted Imaging

**DOI:** 10.1038/s41598-019-40997-6

**Published:** 2019-03-14

**Authors:** Marco Leonetti, Alfonso Grimaldi, Silvia Ghirga, Giancarlo Ruocco, Giuseppe Antonacci

**Affiliations:** 1 Center for Life Nano science @ Sapienza, Isituto Italiano di Tecnologia, Viale Regina Elena, 291, I-00161 Roma, Italy; 2 CNR NANOTEC Institute of Nanotechnology, Via Monteroni, 73100 Lecce, Italy; 3 Dipartimento di Fisica, Universitá “La Sapienza”, Piazzale Aldo Moro, 5, I-00185 Roma, Italy; 4 Photonics Group, Ghent University - imec, Technologiepark Zwijnaarde 15, 9052 Ghent, Belgium

## Abstract

Standard imaging systems provide a spatial resolution that is ultimately dictated by the numerical aperture (NA) of the illumination and collection optics. In biological tissues, the resolution is strongly affected by scattering, which limits the penetration depth to a few tenths of microns. Here, we exploit the properties of speckle patterns embedded into a strongly scattering matrix to illuminate the sample at high spatial frequency content. Combining adaptive optics with a custom deconvolution algorithm, we obtain an increase in the transverse spatial resolution by a factor of 2.5 with respect to the natural diffraction limit. Our Scattering Assisted Imaging (SAI) provides an effective solution to increase the resolution when long working distance optics are needed, potentially paving the way to bulk imaging in turbid tissues.

## Introduction

When illuminated with coherent light, turbid media generate speckle patterns, macular light structures arising from the interference of the scattered or transmitted electromagnetic waves^1–6^. In the bulk of a scattering system, these “*embedded speckles*” show peculiar properties because they typically exhibit a smaller dimension than the illumination point spread function (PSF) of an imaging system^5^. An embedded speckle can indeed reach a size that is independent on the system NA and given by *λ*/2n, with n being the refractive index of the medium and *λ* the wavelength^6–9^.

Recent studies have used the speckle patterns to enhance the focusing or imaging capability of an optical system using opaque layers as “turbid lenses”^4,5,10^. When placed close to the sample, special turbid lenses can indeed yield a subdiffraction resolution^6,11,12^. However, standard turbid lens-based imaging systems, rely on a high intensity speckle grains^13^ generated by wavefront shaping in a “transmission” geometry, which makes them not practical for “*in vivo*” measurements of biological systems.

The use of speckle patterns has further seen extensive use, for example, in blind structured illumination microscopy (Blind-SIM) where an image is reconstructed with an improved resolution by sophisticated deconvolution algorithms^14,15^, typically exploiting the prior knowledge of the sample properties^16–18^. Fluctuation imaging (SOFI)^19^ is another technique that relies on higher-order statistical analysis of temporal fluctuations to improve the resolution. In SOFI, resolution is ultimately limited by the size of the fluctuating item^20^ (typically the fluorescent molecule or a single speckle grain). Nevertheless, the practical limit is much lower due the very high number of frames required to achieve sufficient statistics. In a typical blind-SIM experiment, the speckle grain size is indeed limited by the NA of the illumination and collection optics, which imposes a threshold on the maximum resolution achievable with linear techniques.

In this work, we describe an illumination strategy employing an “opaque mounting medium” (OMM) and a custom deconvolution algorithm to improve the imaging resolution by a factor of ~2.5. Our SAI approach exploits the high spatial frequency content generated by the strongly scattering materials placed in the vicinity of the fluorescent samples under analysis. Moreover, a backscattering (reflection) rather than a typical transmission geometry^5^, is used to enhance the resolution, resulting in an imaging protocol which is particularly advantageous when long working distance optics is needed and transmission geometry is forbidden.

## Results

**Figure 1:**
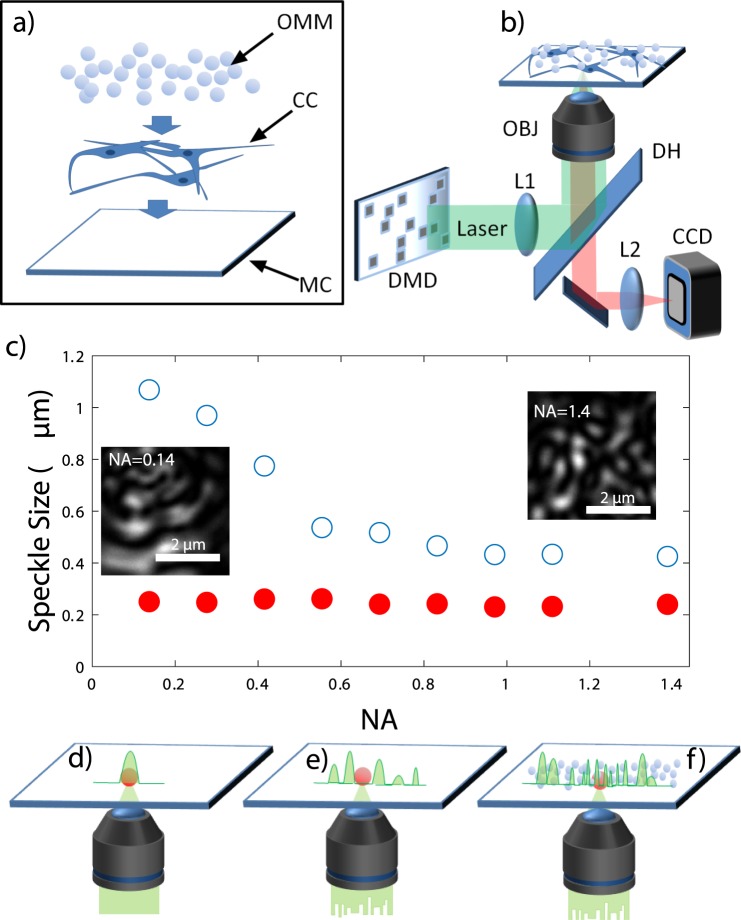
(**a**) A sketch of the opaque mounting medium (OMM). CC: cell culture. MC: microscopy coverslip. (**b**) Experimental Setup. The wavefront of a laser beam (*λ* = 532 nm, laser waist 0.7 mm, divergence < than 0.5 mrad) is modulated by a digital micromirror device (DMD, 1024 × 768 micromirros, pixel size of 13 *μm*) to generate a speckled beam. The active area (50 × 50 micromirrors) of the DMD is imaged with a demagnification factor of 11 at the sample plane through a telescope composed by lens L1 (f = 200 mm) and the illumination/collection objective (OBJ). Fluorescent light from the sample is collected through a dichroic mirror (DH) and imaged on a CCD camera through a lens L2. (**c**) Measured speckle size as a function of the NA. Open circles are relative to a flat mirror sample. Full circles are relative to biological sample covered with the OMM. Panels (d–f) show three possible illuminations configurations. In a standard illumination (panel d), the focus spot size is defined by the illumination NA. In panel (e), the focal spot size is the same as (**d**) but the illumination is speckled due to the input scrambled wavefront. In (**f**), the speckle size is smaller than the objective PSF due to the presence of the OMM.

To obtain speckles of size smaller than the one defined by the illumination geometry, we built an experimental setup shown in Fig. 1. When illuminated by an expanded laser beam, the OMM generates a sample illumination containing high spatial frequencies in the form of speckles of small dimension. This concept is shown in Fig. 1c, where the measured size of the speckle grains (obtained from the FWHM of the autocorrelation function of the intensity profile) are plotted as a function of the effective NA of the illumination beam. To control the illumination NA, we introduced an iris of variable diameter *D* between *L*1 and *DH* and measured the speckle patterns as a function of *D*. On the other hand, the collection numerical aperture was kept constant to provide a nominal spatial resolution of ~180 nm. The experiment has been performed in two configurations. In the first configuration, the size of the speckles generated by the *DMD* + *OBJ* system was measured without introducing disorder (i.e. using a polished mirror as a sample) whilst in the other, speckle size was measured on a biological sample mounted with our OMM (red markers in Fig. 1c). Our results show that when disorder is introduced through the OMM, the speckles size becomes independent on the illumination NA. This concept (depicted schematically in panels 0d-e-f) is at the heart of the Scattering Assisted Imaging (SAI) where the constant dimension of the illuminating speckles is exploited to improve the imaging resolution. In our experimental conditions we retrieved an average speckle size *S* of 240 ± 10 nm.

**Figure 2:**
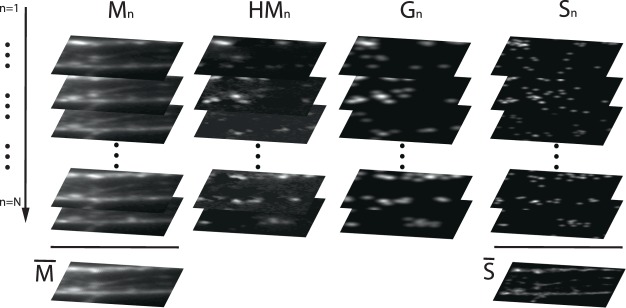
*N* fluorescence images (*M*_*n*_) of a neuron culture stained with Tubulin (see methods) obtained with the illumination patterns *I*_*n*_ are shown in the first column on the left. Adding all contributions, we obtain the average frame $\bar{M}$ (pile bottom). The high intensity part of the fluorescence frame is obtained by subtracting the average frame and considering the positive part of the result. *HM*_*n*_ are reported in the second pile of frames. Applying our gradient descent algorithm, we obtained the *G*_*n*_ (shown in the third column from the left) by minimizing the cost function *F*. The frames shown in right column report the retrieved *S*_*n*_. The high resolution image $\bar{S}$ is obtained by averaging all the *S*_*n*_.

Although the size of speckle grains at the sample plane is defined by the scattering properties of the OMM rather than the illumination optics, convolution with the PSF defined by the finite collection lens imposes a limit on the final imaging resolution of the system. To exceed this limitation, we implemented a custom deconvolution algorithm (Fig. 2 and see methods) relying on the prior knowledge of the speckle size.

Our deconvolution process relies on the localization and the intensity assessment of the generated speckle grains through a gradient descent algorithm that minimizes the differences between the target and the guessed distribution of the speckle grains. Instead of using the whole information, we restricted our analysis to the high intensity part of the signal which is originated by speckle grains of high intensity (HIGs). This approach, which relies on a sparser dataset, has the advantage of providing a much faster and more reliable convergence(see methods and Supplementary Materials), a consequence of the significant number of speckle configurations that excite the *M*_*n*_ fluorescent signal. On the other hand, *HM*_*n*_ is originated by HIGs with intensity higher than the average value. These HIGs are rare and spatially sparse light structures (see Supplementary Materials), thus the deconvolution algorithm requires significantly lower iterations and yields a higher localization accuracy.

**Figure 3:**
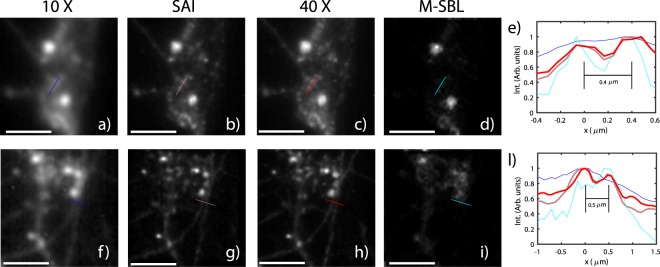
Low resolution (NA = 0.25, nominal resolution *R* = 1.1 *μm*) fluorescence images obtained (**a,f**) obtained by averaging N = 600 fluorescent frames *M*_*n*_. Images obtained with SAI (**b,g**) with N = 600. Representative images (**g,h**) obtained with a high numerical aperture objective (NA = 0.75, nominal resolution *R* = 360 *μm*). Representative images obtained with M-SBL reconstruction algorithms with the same N (**d,i**). Intensity profiles (**e,l**) along the indicated line profiles of the low fluorescence (blue), SAI (pink), M-SBL (cyan) and high NA (red) images. Scale bar is 3 *μm* in (**a–d**) and 6 *μm* in (**f–i**).

Figure 3 shows the results obtained applying SAI to a fixed neuron network sample (see^21^ and methods). In particular, Fig. 3a,f show images from two distinct fields of view obtained averaging *N* = 600 speckle frames measured with a 10 × (NA = 0.25) objective. In other words, these images illustrate the performance of a standard epi-fluorescence microscope where the sample is illuminated with a uniform illumination. In turn, Fig. 3b,g show images obtained using the SAI approach across the same field of view. As a reference, we further report images obtained with a high numerical aperture (NA = 0.75) objective (Fig. 3c,h). Intensity profiles reported in Fig. 3e,l) show a resolution of 0.4 *μm* with an objective which has a nominal resolution of 1.1 *μm* thus providing a resolution enhancement of 2.75. As a further comparison, the same frames were analyzed with multiple sparse Bayesian learning (M-SBL)^16^ based on compressive sensing^22,23^, which is one of the most promising algorithms for the blind-SIM image deconvolution. We adapted a recent version of the algorithm^24^ to our dataset (Fig. 3d,i). While the algorithm catches a resolution similar to that obtained with SAI, the samples appear altered with respect to the ground truth. This difference may be due to the requirement of a sparse fluorescence arrangement for the M-SBL^25^. The improved resolution by SAI is further confirmed by measuring nanometric beads (see Supplementary Materials).

**Figure 4:**
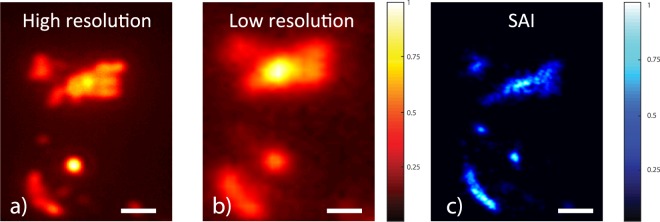
Images of amyloid plaques in a 250 *μ*m brain slice obtained in a standard configuration with a high NA = 0.75 (**a**) and a low NA = 0.25 objective (**b**). (**c**) Reconstructed SAI image after deconvolution obtained with the low resolution objective lens. SAI resolution was measured to be 0.60 ± 0.04 *μm* against a theoretical resolution of 1.10 *μm* given by the objective. We note that despite a measured speckle size *S* of 350 nm, we retrieve a lower resolution due to the strong sample autofluorescence which is decreasing the effective signal to background ratio in the tissue. The bars are 2 *μm*

In the results described above, the scattering was introduced by modifying the optical properties of the mounting medium, thus somehow acting on the sample (even without affecting the fluorescence distribution). To demonstrate the effectiveness of the technique on a naturally scattering sample, i.e. in a condition in which no further action is needed during the preparation stage, we repeated the measurements using semi-transparent brain slices derived from a mouse model of the Alzheimer’s disease containing labelled Amyloid Beta plaques (see Supplementary Materials). High resolution (obtained with NA = 0.75), low resolution (obtained with NA = 0.25) and SAI images (obtained with the same NA = 0.25 objective) of the Amyloid Beta plaques are reported in Fig. 4a–c respectively. Results demonstrate the feasibility of SAI imaging even in biological tissues that naturally introduce a wide range of **k**-vectors from intrinsic scattering. It needs to be noted that the improved resolution was obtained imaging plaques located at the surface of the brain slices. When imaging deeper (~100 *μ*m) within the tissues, the reduced signal to noise ratio significantly affected the system performance as a consequence of the higher uncertainty in the speckle localization.

## Discussion

In summary, SAI provides an increased resolution without the need of high power laser beams or pulsed sources, while blinking or photo switchable fluorescent chemical compounds are unnecessary. Compared to standard SIM^15,26^ and other blind-SIM approaches, our strategy is not sensitive to optical aberrations nor experimental errors degrading the illumination patterns. Furthermore, SAI does not need many priors^14,17^ other than the knowledge of the speckle size and the assumption that the sum of the illumination patterns is uniform. With respect to the most modern algorithms such as joint support recovery^16,25^, our approach is less prone to the requirement of sample sparsity. Another algorithm for speckle-based image reconstruction is S-SOFI^20^ which however improves the imaging resolution with respect to standard wide field techniques by a factor of ~1.6.

The relative simplicity of the experimental setup makes SAI desirable as it can be applied to basic fluorescence imaging schemes where fluorescent molecules lie on the surface of a strongly scattering material. Since the resolution limit is driven by the size of the speckle grain rather than the system NA, the proposed technique shows its maximum advantage in experiments where high resolution is needed together with a long working distance, a condition that is currently forbidden by the physical NA of the illumination and collection optics^27,28^. Involving minimal optical power, SAI can be a powerful tool for the investigation of systems with low damage threshold, and may be easily exported to *in-vivo* investigation of sensible tissues such as the human retina.

## Methods

### Size of the speckle in the sample plane

As specified in the results section, the experiment was performed in two configurations. In the first, we measured the size of the speckles generated by the *DMD* + *OBJ* system without disorder, i.e. using a polished mirror as sample. As a result, the size of the speckle grains was purely determined by the numerical aperture of the objective. In the second configuration, we measured the speckle size on a biological sample, which was mounted with our OMM (red markers in Fig. 1c). Here, the size of the individual micromirrors is expected to play no role in determining S, as the size of their image in the objective plane (13 *μm*/11 = 1.1 *μm*) is comparable with the objective resolution. Moreover, the size of the speckle grains is determined by the properties of the material. A simple numerical simulation, performed by summing random plane waves described by the appropriate eigenvector, allows to retrieve the speckle size in the material by knowing the average refractive index (see Supplementary Materials). In both cases, the coherence of the laser beam affects the speckle contrast. In particular, a laser with a coherence length of at least few centimeters is necessary to ensure sharper speckles.

### Experimental setup for speckle measurement and Opaque Mounting Medium

The proposed configuration is based on a standard imaging scheme in which the illumination is modulated by a DMD generating a scrambled (speckled) wavefront. To exceed the speckle size limit defined by the collection-illumination optics, we exploited the OMM. In particular, we embedded a (barely scattering) cell culture in the strongly scattering mounting medium. The opaque mounting medium is a transparent gel embedding the sample (typically a cell culture or a tissue slice) stained for immunofluorescence experiments^29^.

### Reconstruction Algorithm

In general the fluorescence pattern ${M}_{n=1\mathrm{....}N}$ retrieved with an unknown speckle illumination generated by many successive patterns ${I}_{n=1\mathrm{....}N}$ is given by

1 $${M}_{n}=({I}_{n}\rho )\cdot h+\varepsilon $$

where *ρ* is the (fixed) distribution of the fluorophores and *h* is the PSF of the collection optics while $\cdot$ is the convolution product operator and *ε* is a noise term. The *n*_*th*_ illumination pattern is obtained by randomly orienting the DMD micromirrors, obtaining a total of *N* fluorescence images. By averaging over all the *N* speckle frames we obtain the average fluorescence frame $\bar{M}$. By subtracting to each frame the average frame and taking the positive part (indicated by^+^) of the signal we isolate the part of the signal defined by $H{M}_{n}={({M}_{n}-\bar{M})}^{+}$, which is originated by speckles of high intensity. Taking into account Eq. 1, we obtain

2 $$H{M}_{n}={(({I}_{n}\rho )\cdot h-\bar{M})}^{+}+\varepsilon .$$

In fully developed speckles, high intensity grains are (on average) rare and sparse because the intensity probability density function is exponentially decreasing (see Supplementary Materials and^7^). Indeed we exploit the *HM*_*n*_ dataset to extract information about the underlying fluorescence distribution. Our hypothesis is that *HM*_*n*_ is generated by a superposition of Gaussian light structures of FWHM *S* convoluted with the collection optics PSF:

3 $$H{M}_{n} \sim {G}_{n}=({S}_{n})\cdot h+\varepsilon $$

with

4 $${S}_{n}=\sum _{k=\mathrm{1:}{K}_{n}}\,{S}_{nk}=\sum _{k=\mathrm{1:}{K}_{n}}\,{A}_{nk}\,\exp ((-\,{({\bf{r}}-{{\bf{R}}}_{nk})}^{2})/\mathrm{(2}{S}^{2}))$$

where **r** is the coordinate vector in the image plane and *A*_*nk*_, *R*_*nk*_ and *K*_*n*_ are the intensity, center and total number of the speckle grains respectively that have to be determined.

To find the best distribution of Gaussians producing the target signal *HM*_*n*_ we implement a proximal gradient descent algorithm^30,31^ minimizing the cost function

5 $$F={|H{M}_{n}-{G}_{n}|}^{2},$$

which measures the distance between the target *HM* and *G*_*n*_. Being *A* the area of the fluorescent image calculated as the fraction of pixels above the noise level and ${A}_{speckle}=\pi {(S\mathrm{/2)}}^{2}$ an estimate of the area of the single speckle grain, the starting configuration is initialized with a number of speckles equal to ${K}_{n-start}=A/{A}_{speckle}$ which are cast in random fashion in the field of view. In the first quarter of the optimization protocol (thermalization time), the gradient descent algorithm is free to eliminate or add grains thus effectively finely tuning the number of speckle grains. We verified that the particular initial conditions are only slightly affecting the optimization procedure if a sufficient thermalization window is present. Once the pattern *G*_*n*_ is initialized by randomly casting both the intensity *A*_*k*_ and the center *R*_*k*_ of the Gaussians, the algorithm undergoes an optimization process in which a random change of the two parameters is accepted if *F* is diminished. The procedure is repeated for all the acquired frames and the final high resolution image $\bar{S}$ is obtained by averaging all *S*_*n*_ obtained from the gradient descent procedure as shown in Fig. 2.

### Reliability of the Reconstruction algorithm

To characterize the reliability of the reconstruction algorithm we measured the degree of similitude *Q* between the result of two independent gradient descent minimizations *a* and *b* ($Q=\int {{\mathfrak{N}}}_{n}^{a}{{\mathfrak{N}}}_{n}^{b}dr$, where ${{\mathfrak{N}}}_{n}^{a}=\alpha {S}_{n}^{a}$ with *α* a normalization factor chosen such that $\int \,{{\mathfrak{N}}}_{n}^{a}\ast {{\mathfrak{N}}}_{n}^{a}dr=1$). We found that results are very similar (*Q* ~ 0.86) if the gradient descent is performed on the high intensity part of the data (*HM*_*n*_). On the other hand, a degree of similitude of *Q* ~ 0.35 is found if the original dataset *M*_*n*_ is treated in the same manner.

### Primary cortical neurons

Primary neuronal cultures were prepared from B6/129 early post-natal (P0-P1) mouse cortex according the protocol previously described in ref.^21^ of the main paper. Briefly, cortices were isolated from brains and they were dissociated by 20′ incubation in 0.25% trypsin (15090046, Gibco, Thermo Fisher Scientific) at 37 °C, 5′ in 0.03% DNase (000010, Sigma-Aldrich) at RT and mechanically triturated with a fire-polished Pasteur pipette. Cells were plated on poly-l-lysine-coated glass coverslips, and maintained in Neurobasal (21103049, Gibco, Thermo Fisher Scientific) supplemented with 2% B27 (17504044, Gibco, Thermo Fisher Scientific), 1% L-Glutamine 200 mM (59202C, Sigma-Aldrich) and 1% Penicillin-Streptomycin (P4333, Sigma-Aldrich). Cells were cultured in controlled environment, with a humidified atmosphere containing 5% CO_2_ at 37 °CC. Half of the grow medium was changed every 2 days.

### Immunofluorescence assay

After 14 days, neuronal primary cultures were stained for the detection of BetaIII-tubulin. Briefly, dishes were fixed in 4% PFA 15′ and, after 3′ permeabilization in 0.1% Triton X-100 and 1 h blocking in 1% BSA, they were incubated with primary antibody (T2200, Sigma Aldrich, 1:1000 in 0.1% BSA). After 18 h and 3 washes in PBS, secondary antibody (anti-rabbit Alexa Fluor 532, #A-11009, Thermo Fisher Scientific, 1:500 in 0.1% BSA) was added for 45′ and coverslips were then dehydrated by consecutive 2′ washes in increasing doses of ethanol (30-50-70-90%) and then mounted with our opaque mounting medium.

### Opaque mounting medium

Our OMM is realized mixing a standard mounting medium for fluorescence (Agilent-dako fluorescent medium S3023) with Zinc Oxide powder (Zinc Oxide nanopowder <100 *nm* Sigma Aldrich 544906) in a 2 Molar Solution. The OMM backscatters illumination light, generating a contribution with spatial frequencies higher than that of the bare illumination and thus producing smaller speckles. We depose by drop-cast 20 *μ*l of OMM on the microscopy coverslip which is hosting the culture, which is then squeezed with a second microscopy coverglass which is then sealed, thus sandwiching the culture between the coverglass and the OMM.

### Brain Slice preparation

All experiments on animals were conducted in conformity with European Directive 2010/63/EU and the Italian D.lg. 4.05.2014 and all methods were carried out in accordance with relevant guidelines and regulations. One-year old 3xTg-AD mice were euthanized and transcardially perfused with cold Phosphate Buffered Saline (PBS) solution. 300 *μ*m thick slices were obtained with a vibratome. Slices were fixed in a 4% Paraformaldeide solution for 16 hours at 4 °C and then processed for the free-floating immunostaining. Slices were treated with a solution of 70% formic acid for 30′ to reveal antigen and then blocked with 3% goat serum and 0.3% Triton X-100 in PBS for 1 hour; Amyloid Beta-recognizing primary antibody (803001, Biolegend) was added 1:100 in a solution of 1% goat serum and 0.1% Triton X-100 in PBS at 4 C for 16 hours in continuous agitation. After 3 washes in PBS, Goat anti-Mouse IgG (H&L) Coated Fluorescent Nile Red secondary antibody (MFP-0556-5, Spherotech) was added for 1 h and then the last 3 washes in PBS were performed. Stained brain slices were mounted on a slide with a fluorescent mounting medium (Agilent-dako fluorescent medium S3023) and covered with a coverslip.

## Supplementary information

Supplementary Information

## Data Availability

No restrictions on the availability of materials or information is present.
