# Supplementary material for: Scattering Assisted Imaging
Source: Sci Rep. 2019 Mar 14;9:4591. doi: 10.1038/s41598-019-40997-6 (PMC6418275; doi:10.1038/s41598-019-40997-6)
Supplement: Supplementary file 1 — Supplementary Information [file 41598_2019_40997_MOESM1_ESM.pdf]

# Scattering Assisted Imaging, Supplementary Information

Marco Leonetti*[1,2],Alfonso Grimaldi[1], Silvia Ghirga[1,3], Giancarlo Ruocco[1,3], Giuseppe Antonacci[1,4]
[1] *Center for Life Nano science @ Sapienza, Isituto Italiano di Tecnologia, Viale Regina Elena, 291, I-00161 Roma, Italia*
[2] *5 CNR NANOTEC-Institute of Nanotechnology c/o Campus Ecotekne, University of Salento, Via Monteroni, 73100 Lecce*
[3] *Dipartimento di Fisica, Universitá "La Sapienza", Piazzale Aldo Moro, 5, I-00185 Roma, Italia and*
[4] *Photonics Group, Ghent University - imec, Technologiepark Zwijnaarde 15, 9052 Ghent, Belgium.*

## I. SPECKLES PROPERTIES

The SAI technique employs 'embedded speckles' (i.e. speckles generated by scatterers very close to the fluorescence sample) to obtain, on the one hand, a sparse dataset suitable for the deconvolution algorithm, and on the other, a smaller focus than the one dictated by the illumination NA. The increased sparsity of the illumination is resulting from the well known probability density function $P(I/\overline{I})$ of the intensity $I$ in a speckle field: $P(I/\overline{I}) \propto \exp -I/\overline{I}$, (where $\overline{I}$ is the average intensity).

Fig. 1a shows a typical speckle obtained with NA=1.4 (100X Olympus objective) collecting light backscattered from a turbid sample made of ZnO nanopowder (< 100 nm Sigma Aldrich 544906, average layer thickness of 200 $\mu m$). The intensity probability density function (Fig. 1b) follows the behavior predicted from theory. The average speckle size $S$ is measured from the intensity autocorrelation function Full Width at Half Maximum (FWHM). $S$ has been measured in our experimental conditions to be 240 $\pm$ 10 nm. The distribution $P(\delta)$ of the scaled distances between first neighbor intensity maxima ($\delta = D/S$ where D is the distance between the maxima) is shown in panel 1c. Results suggest that the maxima tend to appear well separated with a small $P(\delta)$ , for $\delta < 1$.

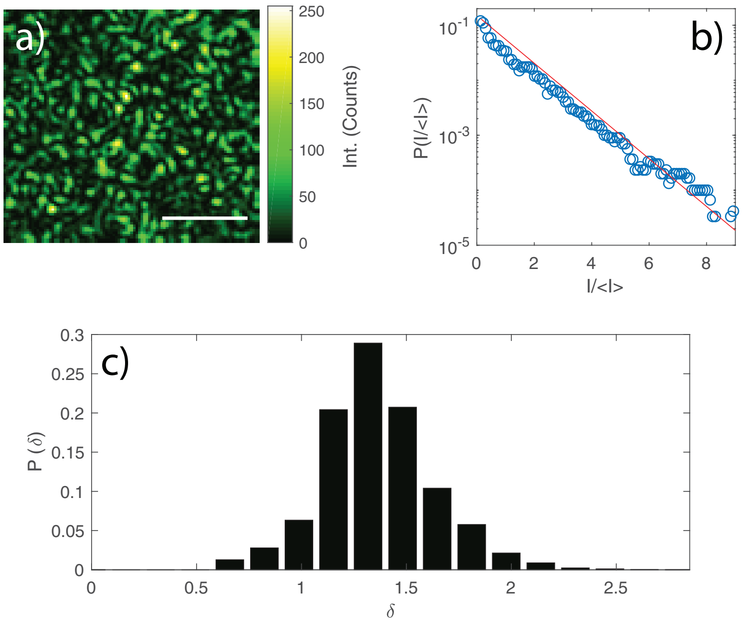


FIG. 1: (a) Typical speckle pattern. Scale bar is 2.5 $\mu m$. (b) Probability density function of the $I/<I>$. Red line represent the fitted curve $P(I/<I>) = (1/<I>)\exp -I/<I>$. (c) Probability of the distance between the first neighbor maxima $P(\delta)$ as a function of $\delta = D/S$.

To estimate speckle size into or in the vicinity of a disordered material, we employed a simplified numerical simulation based of the sum of a sufficient number of plane waves traveling in a random direction ($\mathbf{k} = [k_x k_y k_z]$,$|\mathbf{k}| = 2\pi * \mathfrak{n}/\lambda$, with $\mathfrak{n}$ the refractive index and $\lambda$ the illumination wavelength) and with a random phase delay on a probe axis.

To obtain the fully developed condition, we summed 1000 different plane waves, which resulted in a speckle pattern as the one reported in Fig. 2. The experimental conditions and the properties of the scattering medium (refractive index of the material and the maximum inclination angle) were tuned in the simulation acting on the relevant parameters.

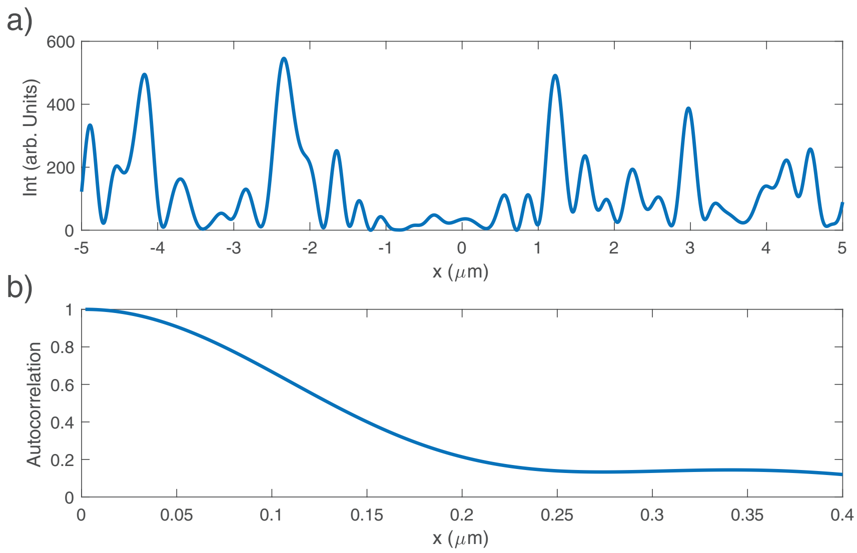


FIG. 2: Typical intensity along x axis from the sum of 1000 plane waves and the correspondent autocorrelation function obtained numerically. Simulation has been obtained with a refractive index of $\mathfrak{n} = 1.615$ which is the one obtained in our OMM. The retrieved speckle size is 224 nm.

The average speckle size is extracted from the autocorrelation of the speckles by measuring the FWHM of the associated intensity profile.

## II. TEST OF THE DECONVOLUTION ALGORITHM ON SIMULATED DATA

To verify the resolution performance of our custom deconvolution algorithm, we performed a numerical experiment. In Fig. 3 we report the reconstructed fluorescence profiles obtained form a sample made of two parallel bars of 50 nm thickness placed at a controlled distance. The fluorescence signal is obtained by overlapping the bars to the speckle patters obtained experimentally ($S$=240 nm) and convolving the emission with the known PSF of the objective (NA =0.25). The results for bars with different distances are reported in Fig. 3a-c. The intensity profiles (white line) show a deep at $X = 0$, which is increasingly pronounced as the bars distance increases. To determine the resolution we used the Rayleigh criterion. Fig. 3d shows a plot of the deep intensity percentage as a function of the bar distance. From these simulations, we extracted a theoretical resolution of 380 $\pm$ 20 nm.

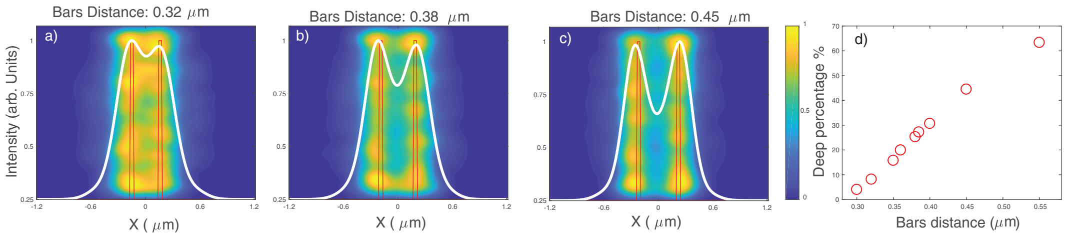


FIG. 3: Reconstructed intensity profiles for different values of the bar distance. The white curve is the intensity profile along the x axis. In red the bars position ground truth.

## III. RESOLUTION ESTIMATION

To estimate the transverse spatial resolution of SAI, we performed measurements on fluorescent beads embedded in both the OMM and in biological tissues. Beads (Speherothec FP-0256-2, 250 nm diameter, dope with Nile Red fluorescent) were measured with a 10X NA=0.25 objective (600 frames). The fluorescence image is obtained by averaging all frames (Fig. 4a,e) while the high resolution image is obtained with the SAI protocol reported in the

main text (Fig. 4b,f) ). As a comparison, we further report the image retrieved on the same field of view with a high resolution objective (40X NA=0.75, Fig. 4c,g). Fig. 4a-d are obtained exploiting the OMM with same protocol described by Fig. 3 of the main paper. Moreover, 4e-h show relative beads in contact with a 300 $\mu$m thick brain slice, in turn reproducing the experimental conditions of Fig. 4 of the main paper. In this case the speckle size is determined by the scattering generated by the biological tissue rather than the OMM, resulting in $S$= 350 nm. The resolution is estimated from the intensity profiles, which are reported in Fig. 4d) and 1h). The OMM enables a resolution of 0.4 $\mu$m while intrinsic scattering of the biological scattering material enables a maximum resolution of 0.6 $\mu$m. The difference between the SAI resolution in the two media is also due to the higher autofluorescence of the former.

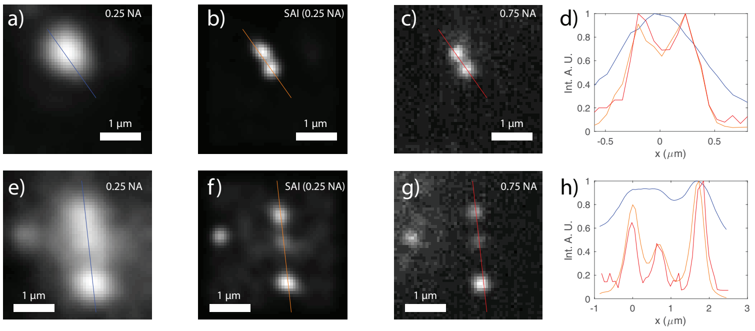


FIG. 4: Fluorescence image of 250 nm beads embedded in the OMM (a-d) or in brain brain tissue (e-h). a) Fluorescence image obtained by averaging N=600 frames with a low resolution objective (10 ×, NA=0.25). b) High resolution SAI image obtained applying the SAI deconvolution on the 600 frames taken with the low resolution objective. c) High resolution image obtained with a 10 ×, NA=0.75 objective. d) Intensity profile along the path highlighted by the colored lines in images a-c. Blue line is from the low resolution image, orange from the SAI Image and red is from the image taken with the high resolution objective. Panels e-g) are taken in the same conditions of a-c with the only difference that the beads are in contact with a 300 $\mu m$ thick brain slice (no OMM present). h) Intensity profile along the path highlighted by the colored lines in images e-g.

## IV. GRADIENT DESCENT ALGORITHM

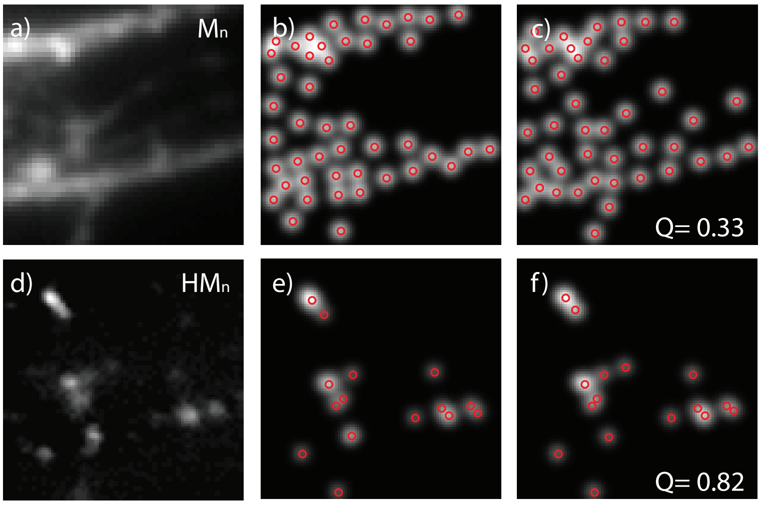


FIG. 5: Comparison of the gradient descent algorithm performance on two different starting frames. Original dataset is reported in panels (a) for $M_n$ and (d) for $HM_n$. The results of two independent executions are reported in panels (b) and (c) for the $M_n$ case and in (e) and (f) for the $HM_n$ case.(b) and (d) are less similar ($Q = 0.33$) then (e) and (f) ($Q = 0.82$).

The reliability of the gradient descent algorithm, which is at the basis of the SAI deconvolution, is affected by the nature of the data to be deconvolved. As described in the main text, we apply the gradient descent optimization to the $HM_n$ i.e. the high intensity part of the data. $HM_n$ frames are composed of rare and typically sparse intensity peaks

embedded in a "dark sea" without information.If $HM_n$ is exploited as target, there is a small set of configurations for HIGs reproducing the data. As a result, the gradient descent algorithm converges much faster and the result exhibits a lower dependency on the random starting conditions. This is confirmed by Fig. 5 in which we compare the result of the algorithm on a typical $M_n$ (raw data) and $HM_n$ data (the high intensity part of the data). The figure shows the original dataset and two independent replicates of the algorithm (with different random starting conditions). The two replicates of the numerical experiments give rise to two significantly different results in the case of $M_n$ (the $Q$ parameter which represents the degree of similarity of the two configurations is 0.33), while they give two almost identical results in the case of $HM_n$ with ($Q$ parameter of 0.82).

The high reliability of the reconstruction algorithm is then confirmed from the data obtained on 100 gradient descent optimizations. The average $Q$ is $0.85 \pm 0.05$ for the $HM_n$ case against the $0.34 \pm 0.12$ for the $M_n$ (error from the statistics over 100 realizations). We also performed the same analysis on 10 different datasets, retrieving an average value of $Q$ always above 0.75 for the $HM_n$ and always lower than 0.40 for the $M_n$.
